# Transfer effects from language processing to visual attention dynamics: The impact of orthographic transparency

**DOI:** 10.1111/bjop.12598

**Published:** 2022-09-18

**Authors:** Antonio Iniesta, María Teresa Bajo, Marta Rivera, Daniela Paolieri

**Affiliations:** ^1^ Mind, Brain and Behavior Research Center (CIMCYC), Department of Experimental Psychology University of Granada Granada Spain

**Keywords:** bilingualism, lexical, local attention, phonological, transparency, writing

## Abstract

The consistency between letters and sounds varies across languages. These differences have been proposed to be associated with different reading mechanisms (lexical vs. phonological), processing grain sizes (coarse vs. fine) and attentional windows (whole words vs. individual letters). This study aimed to extend this idea to writing to dictation. For that purpose, we evaluated whether the use of different types of processing has a differential impact on local windowing attention: phonological (local) processing in a transparent language (Spanish) and lexical (global) processing of an opaque language (English). Spanish and English monolinguals (Experiment 1) and Spanish–English bilinguals (Experiment 2) performed a writing to dictation task followed by a global–local task. The first key performance showed a critical dissociation between languages: the response times (RTs) from the Spanish writing to dictation task was modulated by word length, whereas the RTs from the English writing to dictation task was modulated by word frequency and age of acquisition, as evidence that language transparency biases processing towards phonological or lexical strategies. In addition, after a Spanish task, participants more efficiently processed local information, which resulted in both the benefit of global congruent information and the reduced cost of incongruent global information. Additionally, the results showed that bilinguals adapt their attentional processing depending on the orthographic transparency.

## BACKGROUND

Reading and spelling have been studied extensively to understand how people identify, process and decode different types of words or strings of letters. Several theories have been proposed to explain the mechanism of reading (e.g. see Rayner & Reichle, 2010 for a review). However, one of the models that has received the most attention is the dual‐route cascaded (DRC) model of word identification (Coltheart, 1978; Coltheart et al., 1979, 2001; see also the connectionist models: Harm & Seidenberg, 1999, 2004; Plaut et al., 1996; Seidenberg & McClelland, 1989). According to the DRC model, reading and spelling are processed through two pseudo‐independent routes: the non‐lexical route, which uses phoneme‐to‐grapheme correspondences for letter‐by‐letter word processing, and the lexical route, which retrieves the spelling of the word directly from the orthographic lexicon (Grainger & Ziegler, 2011). Although research on writing is scarce in comparison with reading (Graham et al., 2006), the similarity in the processes assumed to underlie reading and writing has caused dual phonological vs. lexical processing to be extended to writing to dictation (Bonin & Méot, 2002; Delattre et al., 2006; Rapp et al., 2002). Indeed, this dissociation between the two writing processing routes has been reported for patients with acquired dysgraphia (Barry, 1994; Behrmann & Bub, 1992; Goodman & Caramazza, 1986; Tainturier & Rapp, 2000), with a double dissociation between lexical dysgraphia (Hatfield & Patterson, 1983) and phonological dysgraphia (Shallice, 1981).

**FIGURE 1:**
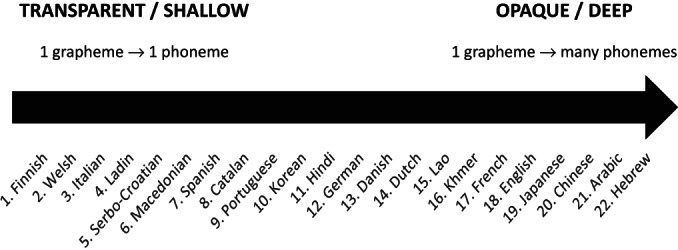
Orthographic transparency across some languages in the world (adapted from Liu & Cao, 2016 and Perfetti & Dunlap, 2008).

The orthographies in alphabetic systems differ in terms of the degree of consistency in the relation between graphemes and phonemes (Schmalz et al., 2015). Importantly, orthographies like Spanish and Italian have a high degree of consistency since, in most cases, they have a one‐to‐one relationship between sounds and letters, and each letter corresponds consistently to one specific phoneme. However, in orthographies such as English or French, the relationship between letters and sounds is not consistent, and there are more one‐to‐more than one‐to‐one options (Ziegler et al., 1997). Grapheme–phoneme consistency determines the degree of transparency, and it varies across languages. Figure 1 represents the differences in terms of transparency across languages as a continuum based on internal regularity. Orthographies that contain words with simple and consistent phoneme–grapheme relations are classified as transparent or shallow orthographies. In contrast, if the phoneme–grapheme relations are ambiguous, with multiple options, the orthographies are classified as opaque or deep (Seymour et al., 2003).

The relevance of orthographic transparency is based on its subsequent impact on linguistic processing (orthographic depth hypothesis; Frost, 1994, 2012; Katz & Frost, 1992). Particularly when learning to read, transparent orthographies mostly engage phonological processing because the very few existing grapheme–phoneme inconsistencies do not require retrieval of the word from the lexicon. In contrast, deep orthographies involve many words that do not follow regular phonological–orthographical rules, and lexical processing is essential to retrieve the correct pronunciations of the words (Bolger et al., 2005; Glushko, 1979; Seidenberg & McClelland, 1989; Seymour et al., 2003; Ziegler & Goswami, 2005). Supporting these assumptions, studies focusing on phonological awareness and lexical access abilities have shown that they are modulated by the transparency of the language (Frost, 1994; Frost et al., 1987; Ziegler et al., 2010). In transparent languages, phonological awareness is acquired and automatized faster than in opaque languages (see Goswami et al., 2005; Patel et al., 2004). Conversely, rapid automated naming (RAN, a standard measure of lexical access) is a more influential predictor of reading performance in opaque languages than in transparent languages (e.g. Caravolas et al., 2012; Moll et al., 2014). The orthographic depth hypothesis (Frost, 1994; Frost et al., 1987) has also been supported by experiments using event‐related potentials (ERPs). For example, the specific component (N320) related to grapheme–phoneme conversion appears more consistently in transparent orthographies (Proverbio et al., 2004; Simon et al., 2006), while components related to lexical integration (N400) are more evident in opaque orthographies (Koester et al., 2007).

**FIGURE 2:**
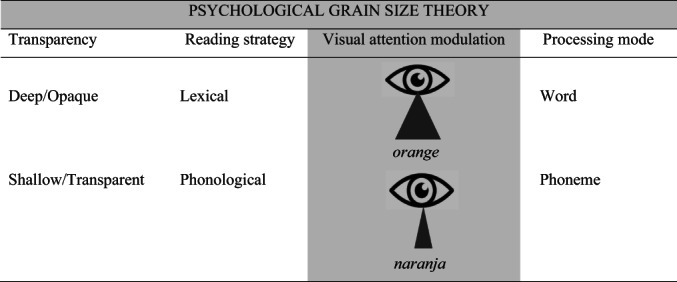
The psycholinguistic grain size theory. The representation of the reading strategies and the modulation of the processing mode (visual attention) depends on the orthographic transparency and the grain size used during reading (adapted from Lallier & Carreiras, 2018).

Both phonological and lexical processing strategies require the contributions of auditory and visual processes (Zoubrinetzky et al., 2014). The role of visual attention in reading has received little interest (Goswami, 2015), and its role in writing has received even less. From another theoretical perspective, orthographic transparency has been proposed to have an impact on the attentional processing window or grain size of processing, particularly during reading acquisition. The psychological grain size theory (PGST; Ziegler & Goswami, 2005) proposes that differences in lexical and phonological processes due to the transparency/opacity of orthographies are associated with differential processing grain size (coarse vs. fine) related to differential attentional windowing (whole words vs. individual letters) (see Figure 2). Using eye‐tracking measures, readers of orthographically opaque languages have been shown to use larger orthographic sequences for analysis than readers of transparent orthographies, who use smaller reading units (Rau et al., 2014, 2015). Indeed, larger visual windows have been related to better performance in irregular word reading (Bosse & Valdois, 2009; Lobier et al., 2013) and to weaker length effects (van den Boer et al., 2013), suggesting a relationship between visual attention and the lexical procedure of reading. Cross‐linguistic data have shown that the number of visual elements that people can process simultaneously is associated with reading speed in opaque languages, while in transparent languages, this association is not observed (Adelman et al., 2010; Awadh et al., 2016; Lallier et al., 2018; Marzouki & Grainger, 2014). Similarly, studies with bilingual children have suggested the potential modulation of visual attention by the transparency/opacity of the languages in which bilinguals are reading (Lallier et al., 2013, 2014, 2016; Valdois et al., 2014). Thus, eye‐tracking studies with German (transparent)–French (opaque) bilinguals (de León Rodríguez et al., 2015, 2016) have shown that the location of the first fixation was closer to the beginnings of the words when reading in a transparent language than when reading in an opaque language, suggesting again that reading strategy is modulated by language transparency, with smaller grains (more local) in transparent languages and larger grains (more global) in opaque languages.

The processing of writing has also been assumed to vary with orthographic depth (e.g. Delattre et al., 2006; Houghton & Zorzi, 2003; Rapp et al., 2002). However, no specific data support this assumption, and it is critical to carry out cross‐linguistic research in this direction (Miceli & Costa, 2014). The main objective of our two experiments was to directly explore whether the use of transparent or opaque language during writing‐to‐dictation tasks would have an impact on attentional windowing. In doing this, we aimed to extend the evidence from reading theories (the orthographic depth hypothesis and the psycholinguistic grain size theory) to word writing in monolingual (using a between‐group design; Experiment 1) and bilingual adults (using a within‐participants design; Experiment 2). It is particularly important to explore the hypotheses of the orthographic depth and grain size theories in the adult population, since these theories were proposed for reading acquisition, and it also becomes mandatory to investigate the dynamics of processing in adults to better conceptualize writing processes.

First, we wanted to test whether writing‐to‐dictation was modulated by lexical or phonological variables, depending on the orthographic depth of the language involved. For this purpose, we decided to explore whether different linguistic variables (i.e. frequency, length, age of acquisition, concreteness and number of orthographic neighbours) differentially affect writing production depending on the language employed (e.g. Norton et al., 2007) as a consequence of the more phonological vs. lexical processing associated with the orthographic transparency of the language used during dictation tasks.

**FIGURE 3:**
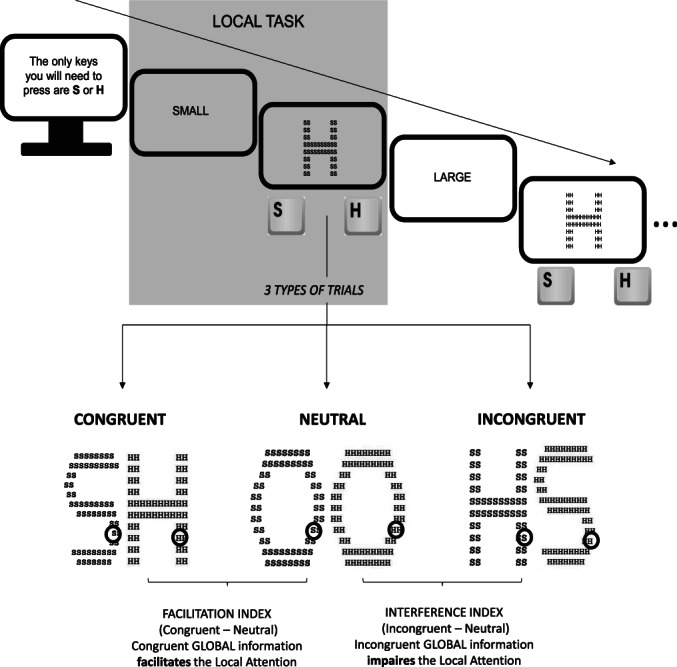
Local trial in the local–global task, including examples of the 3 conditions (congruent, incongruent and neutral) used to calculate the facilitation and interference indexes.

Second, we aimed to investigate whether the type of processing during writing‐to‐dictation (phonological or lexical) would have an impact on the size of the attentional window (Adelman et al., 2010; Awadh et al., 2016; Grainger & Ziegler, 2011; Marzouki & Grainger, 2014; Rau et al., 2014, 2015). To explore this transfer effect, we decided to employ the global–local attentional task (Navon, 1981), which consists of a large letter composed of small letters; the participants had to identify the large (global task) or small element (local task) depending on the instructions (see Figure 3). Recently, it has been proposed that the local task has a connection with lexical and sublexical processing strategies during reading. Franceschini et al. (2021) administered consecutive local trials to induce a ‘local mode’ of processing and consecutive global trials to induce a ‘global mode’. Their results showed that after the induction of a local mode, reading irregular words (which required lexical processing) was significantly slower than reading regular words (which required phonological processing). This pattern suggests a transfer between the local attentional task and the reading strategy. Indeed, the compositional‐local and holistic‐global continuum has also been proposed in several fields, such as number processing (e.g. Pletzer et al., 2021), syntactic processing (McClelland & Patterson, 2002; Pinker & Ullman, 2002) and creativity (Zmigrod et al., 2015), among others.

In our experiments, we decided to explore transfer effects in the reverse direction, that is from the writing task to the visual attention task (global–local task). Although the global–local task has multiple versions, the inclusion of congruent, incongruent and neutral conditions seems to be particularly appropriate (Soriano et al., 2018). In the congruent condition, the large and small letters were the same (e.g. the S letter was composed of small S letters). In the incongruent condition, the large letter was composed of different small letters (e.g. the H letter was composed of small S letters). In the neutral condition, the large letter (H or S) was formed by small circles (O) in the global task, considering that O was never a plausible response (see Figure 3). With this particular design, it is possible to calculate facilitation and interference indexes. Importantly, in the local task, facilitation (the difference between congruent and neutral trials) and interference (the difference between incongruent and neutral trials) come from congruent or incongruent global elements (see Figure 3). Hence, when participants are instructed to focus their attention on the local elements (following the example), the facilitation and interference indexes yield information on the degree to which global information affects local processing (or vice versa, when participants are instructed to focus their attention on global elements).

Although facilitation and interference indexes could be explored when attention is directed to local and global elements (local and global tasks), several reasons suggest that it is pertinent to focus on the local task. First, global processing is prior to local processing; that is, visual information is processed hierarchically, and global processing seems to be a necessary first stage in perception (Navon, 1977, 1981), so the effects from global to local are more robust than those from local to global. Indeed, incongruent global letters have repeatedly been shown to interfere with performance in the local tasks, and congruent global letters have been shown to facilitate performance in the local task. However, in the global task, the results are less robust, and the pattern of interference, especially facilitation from incongruent and congruent local elements, is less consistent (e.g. Mottron et al., 2003; Yovel et al., 2005; see Goldstein‐Marcusohn et al., 2020). Second, regarding the transfer between language and attention, local attention has been shown to have a differential effect on lexical vs. phonological processing, whereas global induction did not have any differential effect on lexical and phonological processing strategies (Franceschini et al., 2021).

## EXPERIMENT 1: SPANISH AND ENGLISH MONOLINGUALS

We asked monolingual participants to carry out a writing‐to‐dictation task and then perform a visual attention task (global–local task). To explore the effect of language transparency, English and Spanish monolingual groups were addressed, considering English as an opaque orthography and Spanish as a transparent orthography. Writing‐to‐dictation tasks have shown high sensitivity to linguistic variables (Bonin et al., 2015). We expected to find evidence of lexical or phonological processing in the writing‐to‐dictation task depending on language transparency. That is, the more extensive use of phonological processing expected in transparent orthographies (i.e. Spanish) relative to opaque orthographies (e.g. English) should make them more susceptible to phonological variables, such as word length or the number of orthographic neighbours (Burani et al., 2007), than to lexical variables, such as word frequency, age of acquisition (AoA) or concreteness. In contrast, the extensive use of lexical processing in opaque orthographies (i.e. English) should make them more sensitive to lexical than phonological factors (Bonin et al., 2002, 2004; Burani et al., 2007). In general, the influence of all these variables on writing performance has been demonstrated across different orthographies (e.g. Bonin & Fayol, 2000; Delattre et al., 2006; González‐Martín et al., 2017; Pinet et al., 2016). Therefore, we expected that these variables would have some influence on writing performance. An advantage of writing‐to‐dictation tasks is that they provide an early and a late measure of writing processes. Thus, two temporal moments can be explored separately; the time needed to initiate writing, and the time spent writing the word (Iniesta, Paolieri, et al., 2021; Muscalu & Smiley, 2018); therefore, it is possible to evaluate the time course of the processing. Hence, while we expected that the effects of our variables would affect late measures of writing (i.e. the rest of the word), analysing the first key stroke (an early measure of writing) would allow us to find evidence of whether the first access to the representation was lexically or phonologically biased (see Baus et al., 2013).

Our second focus was on the transfer effects from the linguistic to the attentional task. We hypothesized that phonological processing in a transparent orthography (Spanish) would activate local attentional processing, while global processing would be induced by lexical processing in an opaque orthography (English). We explored these hypotheses by looking at the pattern of facilitation and interference effects when participants performed the global–local attentional task (Navon, 1981; Soriano et al., 2018). Therefore, we expected that when participants performed the local tasks after the Spanish writing task (therefore introducing a bias towards phonological processing and smaller grain sizes), we would find local processing to be less influenced by global information, with a possible reduction of facilitation and interference effects from global information. In contrast, when the attentional local task was performed after English (lexical processing and a larger grain size), we found that local processing was more influenced by global information, with greater facilitation and greater interference from global information. This would indicate that processing in English promotes global processing, with the consequence of producing greater facilitation when the preferred global information is congruent with the local information and greater interference when the global information is incongruent with the local information.

## METHOD

### Participants

Twenty‐two Spanish monolinguals from the University of Granada (Spain, mean age: 22.05, SD: 3.22) and twenty‐three English monolinguals from Pennsylvania State University (USA; mean age: 23.56, SD: 3.29) were recruited for this study. A criterion for inclusion was that their proficiency level in English as a second language was below B1 (in the European Language Framework) and that they did not use English (or any other second language) in their normal lives. In this sense, they were classified as functional monolinguals, although they might have had second‐language instruction in their early academic lives. They participated in the experiment in exchange for partial course credits. The Spanish monolingual group completed the Language Experience and Proficiency Questionnaire (LEAP‐Q; Marian et al., 2007) to ensure low levels of English comprehension (*M* = 2.07), reading (*M* = 2.23) and speaking (*M* = 1.74) on a scale from 0 to 10. The Spanish monolingual group reported a 4.71% rate of current exposure to English, whereas the English monolingual group reported a 99.27% rate of current exposure to English. Some studies have shown high correlations between self‐reported and objective proficiency measures (e.g. Marian et al., 2007). All participants had normal or corrected‐to‐normal vision and hearing abilities, and they did not show any language or neurological impairments. They all signed consent forms according to the protocol approved by the Ethical Committee at the University of Granada. A minimum sample size of 42 (21 participants in each group) was required to obtain a large effect size (d = 0.8) based on a priori calculation with the G*Power program for t‐tests (test family) specifying the difference between two independent means (two groups), considering one tail with α = .05, power = .80, and an allocation ration = 1, resulting in a non‐centrality parameter of 2.59 (Erdfelder et al., 1996).

### Materials

#### Global–local task

We implemented the adapted version of the classical global–local task (Navon, 1977), which included congruent, incongruent and neutral trials for global and local tasks (Soriano et al., 2018; see Figure 3), as described in the introduction. Each participant underwent 72 trials in the global task and 72 trials in the local task; that is, there were 24 congruent trials, 24 incongruent trials and 24 neutral trials for the local task and the same distribution for the global task. The order of presentation was randomized, considering tasks (local or global), and conditions (congruent, incongruent and neutral) in a single experimental block.

#### Writing‐to‐dictation task

The participants carried out a writing‐to‐dictation task, with words presented aurally in an emotionally neutral tone. The words were recorded in 26‐bit mono with a frequency of 44,100 Hz and filtered from environmental sounds. To control for the influence of the speaker's gender (Casado et al., 2017), we introduced both native English and native Spanish masculine and feminine voices that appeared randomly and equally across conditions. A total of 178 nouns in English and Spanish were included in two separate blocks (see Table 1 for a descriptive analysis of the experimental material and the Appendix S1 for the complete list of the stimuli). English and Spanish logarithmic frequencies and lengths (number of letters) were computed using NimTools (Guasch et al., 2013). The English AoA was extracted from Kuperman et al.’s (2012) ratings, and the Spanish AoA was extracted from subjective norms (Alonso et al., 2014). English concreteness was searched in the word lemmas rating (Brysbaert et al., 2014) and Spanish concreteness in the EsPal database (Duchon et al., 2013). CLEARPOND (Marian et al., 2012) was used to acquire orthographic neighbour information. As expected, there were no significant differences between language blocks in frequency, *t* (89) = −.170, *p* = .865; length, *t* (89) = −1.628, *p* = .107; AoA, *t* (89) = −1.04, *p* = .301; concreteness, *t* (89) = −1.55, *p* = .126; or number of neighbours, *t* (89) = −1.35, *p* = .182. We also controlled the orthographic similarity (OS; Van Orden & Goldinger, 1994) and normalized Levensthein distance (NLD; Levenshtein, 1966; Schepens et al., 2012) between the selected words and their translations. OS and NLD were computed using NimTools (Guasch et al., 2013). The *t*‐test performed showed no significant differences between language blocks: *t* (89) = −.001, *p* = .999 (OS); *t* (89) = .43, *p* = .671 (NLD).

**TABLE 1:** Characteristics of the experimental stimuli.

Blocks	Frequency	Length	AoA	Concreteness	Neighbours	OS	NLD
English	1.017 (0.567)	6.438 (1.637)	7.415 (2.609)	4.051 (1.140)	2.932 (4.355)	0.471 (0.194)	0.496 (0.211)
Spanish	1.033 (0.636)	6.842 (1.851)	7.019 (2.201)	4.29 (1.188)	2.112 (3.369)	0.471 (0.211)	0.510 (0.211)

*Note*: Mean scores with *SD* in parentheses. Frequency = Logarithmic frequency; AoA = age of acquisition; OS = orthographic similarity; NLD = normalized Levensthein distance.

### Procedure

The participants were tested individually. They were asked to perform the dictation task in their native language. After the dictation task, the participants were asked to perform the global–local task. Thus, for each participant, the experiment consisted of two main tasks that proceeded sequentially: (1) the dictation task in Spanish or English (depending on the group) and (2) the global–local task. There was a break between the two tasks (3 minutes). The experimental section lasted approximately 40 minutes.

The writing‐to‐dictation task was programmed on E‐Prime version 2.0. Each trial started with a fixation point that remained on the screen until the audio stimulus was finished. The target word was presented orally through headphones. When the audio was finished, the participant had to write the target word as rapidly and as accurately as possible. The participants were instructed to press the space bar when they finished writing. Writing times and accuracy were collected from the onset of the stimulus to the first keypress (the first key performance) and from the first keypress to the end of the word (the rest of the word performance) (see Iniesta, Paolieri, et al., 2021; Muscalu & Smiley, 2018). A 30‐second break was introduced in the middle of the task (trial 45). Each language block began with 10 practice trials and 89 experimental words.

The global–local task was also administered via E‐Prime version 2.0 (Psychology Software Tools). At the start of each trial, participants were presented with an instruction cue at the centre of the screen for 500 ms indicating whether they should respond to the large (global task) or small (local task) letter. This cue consisted of a screen with instructions to respond to either the ‘large’ global letter or to the ‘small’ local letters (each letter was presented in Courier New [font], 18‐point [size]). Immediately after the instructions, a single stimulus was presented in the centre of the computer screen (with a dimension of 7 × 4 cm) until the participant responded. The participants sat 65 cm away from the screen. At this distance, the local letters had a vertical visual angle of 0.5 degrees. Large letters subtended a vertical visual angle of 10 degrees (Kimchi, 2015). The participants were reminded at the beginning of the task to identify the target letter by making the keypress corresponding to ‘H’ or ‘S’, depending on the trial. Note that ‘O’ was not a plausible response. They were prompted to answer as rapidly as possible but to try not to make mistakes. The participants underwent 10 practice trials before the experimental task, including examples of all experimental conditions. A 30‐second break was introduced in the middle of the task (trial 72).

## RESULTS

### Writing‐to‐dictation task

First, we analysed response times (RTs) and accuracy in the writing‐to‐dictation task to investigate whether the performance was modulated by lexical and/or phonological variables. Within‐subject data trimming was implemented (Sullivan et al., 2018) in which the RTs above or below 2.5 SD from each participant's mean were eliminated from the analysis (3.31% from the Spanish monolingual group and 3.12% from the English monolingual group). Correlational analyses were then performed for each group (Spanish and English monolinguals), including the RTs and ACC for correct responses (CRs) in the first key and rest of the word performances; lexical frequency, AoA and concreteness as evidence of lexical processing (Bonin et al., 2004); and the number of letters (length) and orthographic neighbours as evidence of phonological processing (Burani et al., 2007).

Table 2 includes Pearson correlations between the variables for the two data sets (writing‐to‐dictation performances and linguistic variables). As expected, performance on the rest of the word (the late measure of writing‐to‐dictation) showed correlations with most linguistic variables in both monolingual groups. Specifically, the RTs for the rest of the word were negatively associated with orthographic neighbours and logarithmic frequency but positively associated with AoA and length in Spanish and English (see Table 2). Note that concreteness showed no significant correlation. However, performance on the first key (the early measure of writing‐to‐dictation) showed critical dissociation between groups. In the English monolingual group, RTs were negatively associated with frequency and positively associated with AoA, whereas in the Spanish monolingual group, RTs were positively associated with word length.

**TABLE 2:** Correlation among linguistic variables and writing to dictation performance in each monolingual group.

	Monolingual groups							
	Spanish				English			
	First key		Rest of word		First key		Rest of word	
	RT	ACC	RT	ACC	RT	ACC	RT	ACC
Frequency	−.107	.180	−.289**	.212*	−.323**	.224*	−.238*	.167
Length	.289**	.124	.900**	−.422**	−.098	−.075	.869**	.104
AoA	.004	.110	.322**	−.297**	.255*	−.033	.547**	−.233*
Concreteness	.192	.032	.057	.156	.091	.085	.160	.114
Neighbours	.034	.143	−.601**	.168	−.064	−.106	−.553**	.103

**p < .01; *p < .05.

Because of the theoretical implications of the dissociation between the two language groups in the first key performance, and considering possible biases of an indirect approach to inferential statistics with respect to the null hypothesis (e.g. Wagenmakers et al., 2015), we decided to implement Bayesian Pearson correlations (Love et al., 2015) to test for the robustness of the significant correlations (length, frequency, and AoA; see Table 3). For this purpose, we used JASP software (JASP Team, 2019). This analysis reports a Bayes factor that represents the strength of the evidence that the data provided for the model under consideration (H1; there is a correlation) in relation to the evidence in favour of the null hypothesis (H0; there is no correlation). We used the default Cauchy prior width of 0.707, and the default Beta prior width of 1 was implemented in the Bayes factor robustness check. The results of the Bayesian procedure yielded the same pattern of results as the descriptive statistics.

**TABLE 3:** Bayesian factor analysis of the critical correlations in both monolingual groups.

Correlations	Spanish group	English group
RT – Length	BF_10_ 5450	BF_10_ 0.199
Evidence for H1. Robustness check	Strong^a^	No evidence
RT – Frequency	BF_10_ 0.216	BF_10_ 14.43
Evidence for H1. Robustness check	No evidence	Strong^a^
RT – Age of Acquisition (AoA)	BF_10_ 0.133	BF_10_ 4.591
Evidence for H1. Robustness check	No evidence	Moderate^a^

^a^ A Bayes factor above 3 is considered to be *moderate* evidence, and a Bayes factor above 10 is considered to be *strong* evidence for the alternative hypothesis (Lee & Wagenmakers, 2014).

### Global–local task

A within‐subject data trimming was implemented (Sullivan et al., 2018) in which the RTs above or below a 2.5 SD from each participant's mean were eliminated from the analysis (3.65% of the items were discarded in the Spanish monolingual group, and 3.17% in the English monolingual group). The RTs for CRs of each type of trial during the local task in the Spanish monolingual group (congruent [M = 859.37 ms; SD = 32.19], incongruent [M = 926.89 ms; SD = 41.46] and neutral [M = 922.04 ms; SD = 34.10] trials) and in the English monolingual group (congruent [M = 784.97 ms; SD = 38.52], incongruent [M = 844.75 ms; SD = 36.33] and neutral [M = 792.85 ms; SD = 34.95] trials) were computed in both facilitation and interference indexes (Soriano et al., 2018). Note that, in Spanish monolinguals, the differences between congruent and neutral (facilitation index) were significant, *t* (21) = −2.304, *p* = .032, but the differences between incongruent and neutral (interference index) were not significant: *t* (21) = .194, *p* = .848. In English monolinguals, we found the reverse pattern, where the differences between congruent and neutral were not significant, *t* (22) = −.264, *p* = .826, whereas the differences between incongruent and neutral were significant, *t* (22) = 4.851, *p* < .001.

**FIGURE 4:**
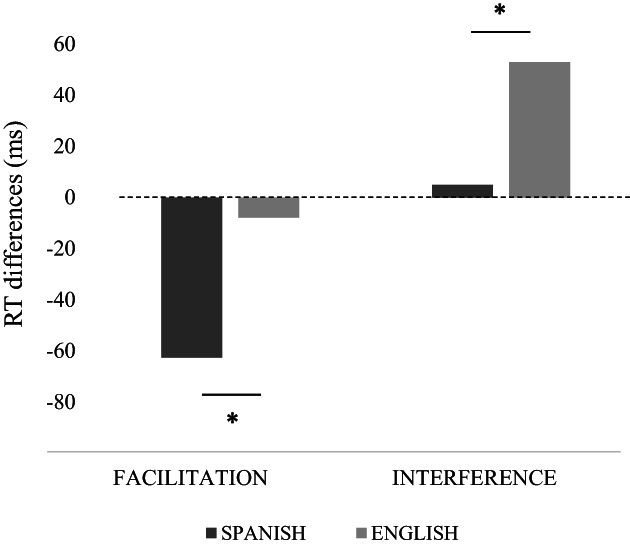
Facilitation and interference indexes in the local task from monolingual groups.

The analyses conducted comparing the facilitation and interference indexes separately between Spanish and English monolinguals (see Soriano et al., 2018) showed greater facilitation for Spanish (M = −62.66 ms) than for English participants (M = −7.88 ms), *t* (43) = −3.532, *SE* = 28.02, *p* = .002, *d* = −0.953, and smaller interference for Spanish (M = 4.85 ms) than for English participants (M = 51.89 ms), *t* (43) = −2.587, *SE* = 24.17, *p* = .016, *d* = −0.771 (see Figure 4).

## DISCUSSION

In Experiment 1, we aimed to explore whether the type of processing (local–phonological or global–lexical) induced by the use of a transparent or opaque language during a writing‐to‐dictation task transferred to a subsequent attentional task, biasing attention towards local features. With this aim, we asked Spanish and English monolingual participants to perform a global–local task after a writing‐to‐dictation task.

First, we explored the effect of certain linguistic variables in English and Spanish writing‐to‐dictation tasks (Norton et al., 2007). As expected, and considering that the participants were adults and consequently experienced writers, the performance in the rest of the word (the late measure of writing‐to‐dictation) showed a general influence of lexical (frequency and AoA) and phonological (length and neighbours) variables in both languages as evidence of the use of both processing strategies (lexical and sublexical) in transparent and opaque orthographies (e.g. Bosse et al., 2003; Folk & Rapp, 2004; Roux & Bonin, 2012; Tainturier et al., 2013). Concreteness did not show any influence on performance, presumably due to the reduced variability of the concreteness values in the items included in the dictation task and in addition to the methodological problems related to the concreteness variable highlighted by Pollock (2018).

Interestingly, the results of the first key (the early measure of writing) showed a clear dissociation between languages: performance in the Spanish monolinguals was modulated by word length, whereas performance in the English monolinguals was modulated by word frequency and AoA. Since word length effects are usually interpreted as the result of phonological processing (Burani et al., 2007) and the frequency and age of acquisition as the result of lexical processing (Bonin et al., 2004), our results support the assumption that language transparency biases processing towards phonological (local) or lexical (global) processing, at least at an early stage in the writing process.

The analysis of facilitation and interference in the local task yielded significantly greater facilitation from Spanish than from English writing. Spanish participants showed that, after the writing task, congruent global information facilitated performance in the local attention task more than English monolinguals after English writing. The differences between languages also extended to the pattern of interference, so that, after Spanish writing, the interference from incongruent information was smaller than after English writing. Although this pattern did not exactly fit our expectations, our results can still be interpreted as supporting our hypothesis. More efficient processing of local information after practicing local (phonological) processing in the Spanish block might have permitted our Spanish participants to use congruent global information to facilitate performance while avoiding the increase in greater interference by incongruent global information when processing local information. This very efficient local processing after the Spanish block contrasted with the seemingly more difficult processing after the English block, where our English participants could not use congruent global information to facilitate performance. However, the incongruent global information impaired their performance, which made their local attention inefficient when using global information. The transfer from processing Spanish words (local bias) to local visual attention was evidenced not only by the larger facilitation effect in the local tasks but also by smaller interference in this task. Overall, the type of processing influenced the size of the attentional window (e.g. Awadh et al., 2016; Grainger & Ziegler, 2011; Rau et al., 2014, 2015).

## EXPERIMENT 2: SPANISH–ENGLISH BILINGUALS

Bilinguals are readers and writers of two or more languages that might differ in their level of transparency. Some studies support the idea that bilinguals adapt their processing to the opacity/transparency of the language they are using (Buetler et al., 2014; Das et al., 2011; Jamal et al., 2012; Oliver et al., 2017; Rau et al., 2014, 2015; Tierney & Nelson, 2009). In addition, this adaptation has been proposed to have an impact on the grain size of processing (coarse vs. fine), which is related to differential attentional windows (whole words vs. individual letters). Bilinguals would use a coarser‐grained process when using the language with a deeper orthography (i.e. English) and a finer‐grained process when using the language with a shallower orthography (i.e. Spanish) (see the grain size accommodation hypothesis; Lallier & Carreiras, 2018).

Some evidence supports the grain size accommodation hypothesis in bilinguals. For example, Lallier et al. (2014) showed an advantage in reading words over pseudowords in bilingual children when the task was performed in their opaque language (French). However, this difference was not evident in their shallower language (Spanish), suggesting that the children used a finer‐grained reading strategy. Similarly, evidence with eye‐tracking showed the location of the first fixation in different positions of the word depending on the transparency, suggesting again that the reading strategy is modulated by the language transparency, with smaller grains (more local) in transparent languages and larger grains (more global) in opaque languages (de León Rodríguez et al., 2015, 2016).

Critically, a previous study on writing‐to‐dictation suggested differences in the processing strategy (lexical vs. phonological) and the grain size of processing depending on the language transparency (Iniesta, Paolieri, et al., 2021). That is, bilinguals committed qualitatively different errors in English and Spanish during a writing‐to‐dictation task involving words with polyvalent graphemes (a sublexical/phonological manipulation in which one phonological representation has two orthographic specifications, for example, /b/for both the graphemes v and b). During the English block (lexical processing), inconsistent grapheme–phoneme mappings induced a more generalized type of error (the errors were distributed across all possible letters composing the word). In contrast, during the Spanish block (phonological processing), the type of error was more specific in the sense that the errors were mainly present on the polyvalent grapheme, indicating that the grain size of the processing could be modulated by transparency.

The aim of Experiment 2 was to seek further evidence about the transfer effects from differential language processing (writing‐to‐dictation) depending on the opacity/transparency of the language over the attention task (global–local task) to explore bilinguals' capacity to adapt their processing to the opacity/transparency of the language they are using. In doing this, we aimed to extend the evidence from reading theories (the orthographic depth hypothesis, the psycholinguistic grain size theory, and the grain size accommodation hypothesis) to word‐writing production in bilingual adults. We sought to replicate the pattern of linguistic and visual attention effects in the subsequent attentional task (local efficiency) obtained in Experiment 1, but in this experiment, language transparency was manipulated within the participants, since they were bilinguals of opaque–transparent (English and Spanish) orthographies. Within‐subject designs provide undeniable advantages by avoiding all sorts of potentially confounding variables across two different sets of participants.

Importantly, we expected that bilingual participants would be able to adapt their processing styles to the transparency of the language used for the writing task, which would in turn be reflected in their attentional type of processing (e.g. de León Rodríguez et al., 2015, 2016). Thus, we expected that, when participants performed the linguistic task in Spanish, we would find evidence that the local efficiency we found in Spanish monolinguals (Experiment 1) while performing the writing‐to‐dictation task in English would result in inefficient local processing, replicating the English monolinguals (Experiment 1).

## METHOD

### Participants

Twenty‐three L1‐Spanish and L2‐English bilingual students (mean age: 21.5, SD: 4.43) from the University of Granada (Spain) participated in the study. They were native Spanish speakers and Spanish‐dominant but with a high proficiency level in English. They had obtained an official English qualification in the previous 2 years before the present experiment (Level B2) and participated in the experiment in exchange for partial course credits. The data of these participants were extracted from previous research whose objective was to study orthographic coactivation in bilingual writing production. All participants had been exposed to English for more than 11 years (M = 13.14), and their self‐rating proficiency was greater than 7 (on a scale from 0 to 10) for understanding (M = 8.05), speaking (M = 7.41) and reading (M = 7.77) in English, as assessed by the Language Experience and Proficiency Questionnaire (LEAP‐Q; Marian et al., 2007). All participants had normal or corrected‐to‐normal vision and hearing abilities, and they did not show any language or neurological impairments. They all signed consent forms according to the protocol approved by the Ethical Committee at the University of Granada. A minimum sample size of 19 participants was required to obtain a large effect size (*d* = 0.8) based on a priori calculation with the G*Power program for t‐tests (test family), specifying the difference between two dependent means and considering one tail, with α = .05, power .95 and the result of a non‐centrality parameter of 3.48 (Erdfelder et al., 1996).

### Materials and procedure

Participants in the bilingual group were asked to complete the global–local letter task and the writing‐to‐dictation task, as were the monolinguals from Experiment 1. After the dictation task, the participants were asked to perform the global–local task. However, in this experiment, the dictation task was composed of two blocks (Spanish and English) administered separately and counterbalanced across participants. For each participant, the experiment consisted of a number of tasks that proceeded sequentially: (1) the dictation task in either Spanish or English, (2) the global–local task, (3) a new dictation task in the alternative language (Spanish or English) and (4) the global–local task. All tasks were performed in individual sessions that lasted <1 h. The materials were the same as in Experiment 1. The participants completed the global–local task after each language block of the writing‐to‐dictation task. To ensure that there were no practice or order effects, we compared the overall times in the local–global task after Spanish as the first block (the first time that the participant carried out the task; 12 participants) vs. Spanish as the second block (the second time that the participant carried out the task; 11 participants), and the overall times in the local–global task after English as the first block (11 participants) vs. English as the second block (12 participants). We did not find differences either in Spanish (*t* (21) = −.753, *p* = .460) or in English (*t* (21) = −.643, *p* = .527).

## RESULTS

### Writing‐to‐dictation task

As in Experiment 1, response times (RTs) and accuracy in the writing‐to‐dictation task were analysed to investigate the effects of lexical and/or phonological variables on writing performance. Within‐subject data trimming was implemented (Sullivan et al., 2018) in which the RTs above or below 2.5 SD from each participant's mean were eliminated from the analysis in each language block (3.15% of the items from the Spanish block, 3.54% from the English block). Correlational analyses were then performed separately for each language block (bilingual‐English and bilingual‐Spanish blocks), including the RTs and ACC for correct responses (CRs) in the first key and the rest of the word, lexical variables (frequency, AoA and concreteness; Bonin et al., 2004) and phonological variables (length and orthographic neighbours; Burani et al., 2007).

Table 4 includes Pearson correlations between the variables for the two data sets (writing‐to‐dictation performances and linguistic variables). As expected, and replicating Experiment 1, performance on the rest of the word (the late measure of writing‐to‐dictation) showed correlations with most linguistic variables in both Spanish and English language blocks, except concreteness (see Table 4). However, performance on the first key (the early measure of writing‐to‐dictation) showed a critical dissociation between languages. In the English block, the RTs were negatively associated with frequency and positively associated with AoA, whereas in the Spanish block, the RTs were positively associated with word length.

**TABLE 4:** Correlation among linguistic variables and writing to dictation performance in each language block in the bilingual group.

	Bilingual group							
	Spanish‐L1 block				English‐L2 block			
	First key		Rest of word		First key		Rest of word	
	RT	ACC	RT	ACC	RT	ACC	RT	ACC
Frequency	−.123	.197	−.384**	.196	−.461**	.233*	−.325**	.439**
Length	.393**	−.052	.807**	−.327**	−.131	−.045	.754**	−.169
AoA	−.098	.094	.370**	−.308**	.274**	−.150	.488**	−.451**
Concreteness	.145	−.083	−.080	.181	.174	−.203	−.101	−.170
Neighbours	.163	.120	−.508**	.170	−.060	.113	−.455**	.171

**p < .01; *p < .05.

In addition, and replicating the data analysis plan implemented in Experiment 1, we also implemented Bayesian Pearson correlations (Love et al., 2015) to test the relation between RTs to the first key and length, frequency, and AoA because they are theoretically critical for our arguments (Love et al., 2015; See Table 5). The results of the Bayesian procedure yielded the same pattern of results as the descriptive statistics.

**TABLE 5:** Bayesian factor analysis of the theoretically critical correlations in bilinguals.

Correlations	Spanish block	English block
RT – Length	*BF*_*10*_ 165.2	*BF*_*10*_ 0.275
Evidence for H1. Robustness check	Strong*	No evidence
RT – Frequency	BF_10_ 0.254	BF_10_ 3467
Evidence for H1. Robustness check	No evidence	Strong*
RT – Age of Acquisition (AoA)	BF_10_ 0.200	BF_10_ 4.697
Evidence for H1. Robustness check	No evidence	Moderate*

*Evidence to support the alternative hypothesis.

### Global–local task

Within‐subject data trimming was implemented (Sullivan et al., 2018) in which the RTs above or below 2.5 SD from each participant's mean were eliminated from the analysis. The cut‐off was performed for each language block independently (3.11% of the items were discarded after the Spanish block, and 2.95% after the English block).

Following the data processing of the monolingual experiment (Experiment 1), the RTs for correct responses of each type of trial during the local task after the Spanish‐L1 block (congruent [M = 720.94 ms; SD = 36.27], incongruent [M = 811.46 ms; SD = 53.86] and neutral [M = 780.80 ms; SD = 43.92] trials) and after the English‐L2 block (congruent [M = 818.85 ms; SD = 32.61], incongruent [M = 869.72 ms; SD = 32.04] and neutral [M = 827.73 ms; SD = 29.81] trials) were computed in both the facilitation and interference indexes (Soriano et al., 2018). Note that after the Spanish‐L1 block, the differences between congruent and neutral (facilitation index) were significant, *t* (22) = −2.659, *p* = .026, but the differences between incongruent and neutral (interference index) were not significant, *t* (22) = 1.774, *p* = .095. After the English‐L2 block, we found the reverse pattern; that is, the differences between congruent and neutral were not significant, *t* (22) = −.832, *p* = .414, whereas the differences between incongruent and neutral were significant, *t* (22) = 3.278, *p* = .003.

**FIGURE 5:**
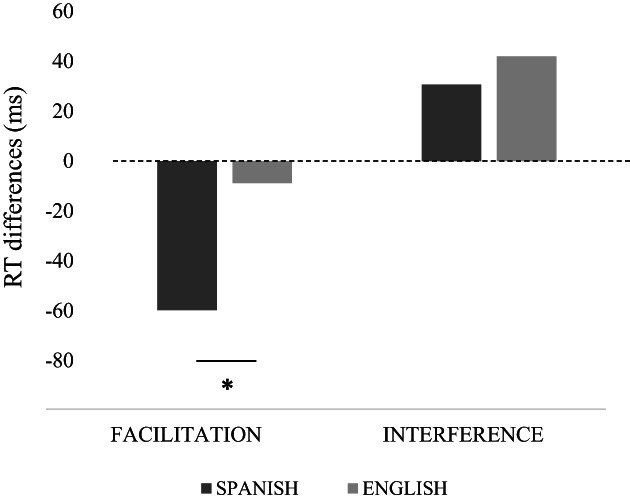
Facilitation and interference indexes in the local task from the bilingual group.

The analyses conducted comparing the facilitation and interference indexes separately after the Spanish and English blocks of the writing‐to‐dictation task (see Soriano et al., 2018) showed larger facilitation after the Spanish block (M = −59.86 ms) than after the English block (M = −8.88 ms), *t* (22) = −3.639, *SE* = 26.29, *p* = .001, *d* = −0.759. However, no differences were found in interference after the Spanish (M = 30.65 ms) or English block (M = 41.99 ms), *t* (22) = −.153, *SE* = 16.56, *p* = .880, *d* = −0.032 (see Figure 5).

## DISCUSSION

The results of Experiment 2 replicated in part those from Experiment 1, providing robustness to the conclusions, since in this last experiment, the within‐subject design reduced potentially confounding variables associated with the participants. Concretely, the performance in the rest of the word (the late measure of writing‐to‐dictation) showed a general influence of lexical (frequency and AoA) but also phonological (length and neighbours) variables in both languages as evidence of the use of both processing strategies (lexical and sublexical) in transparent and opaque orthographies (e.g. Bosse et al., 2003; Folk & Rapp, 2004; Roux & Bonin, 2012; Tainturier et al., 2013). In addition, the results of the first key (the early measure of writing) replicated the dissociation between languages found in monolinguals (Exp. 1) and extended the results to the bilingual population. Performance in the Spanish block was modulated by word length, presumably as a consequence of the predominant phonological type of processing (Burani et al., 2007), whereas performance in the English block was modulated by word frequency and AoA, presumably as a consequence of the bias towards lexical strategies during processing (Bonin et al., 2004). Again, these results indicated that, during the early stage of writing, language transparency biased processing towards phonological or lexical processing in the same direction as the reading theories (Frost, 1994, 2012; Katz & Frost, 1992; Perfetti & Dunlap, 2008; Ziegler et al., 1997).

Similarly, the pattern of transfer between the linguistic writing‐to‐dictation task and the attention task was replicated, also in part, across both experiments. The results of Experiment 2 showed higher local efficacy after the Spanish block than after the English block. As expected, according to Experiment 1, analyses of facilitation and interference on the local task yielded significantly greater facilitation from Spanish than from English writing. However, the interference effects were similar for both languages. More efficient processing of local information after practicing local (phonological) processing in the Spanish block might have permitted our bilingual participants to use congruent global information to facilitate performance to a greater extent than after the English block, where there was no facilitation. In contrast to Experiment 1, this local efficiency in using global information to facilitate performance after Spanish was not accompanied by less interference during local processing. In this experiment, our bilingual participants were not as efficient as Spanish monolinguals in reducing the interference from global information after the Spanish block, as they showed similar global interference effects after the English and the Spanish blocks when processing local information. One possible reason may lie in the fact that our participants were bilinguals since the co‐activation of their two languages might have mitigated possible language differences in facilitation and interference effects. Bilinguals are known to co‐activate their two languages even while processing only one of them (Marian & Spivey, 2003; Paolieri et al., 2010; Van Hell & Dijkstra, 2003), and this co‐activation may act at different processing levels. In addition, because bilinguals read and write in two orthographies, they necessarily use each language less and accumulate less practice in reading and writing than their monolingual counterparts (see the weaker links hypothesis; Gollan et al., 2008). This might explain the less efficient performance of our bilinguals after the Spanish block (Experiment 2) in comparison with our Spanish monolinguals (Experiment 1).

In sum, the results of Experiments 1 and 2 showed more efficient processing of local information after practicing phonological processing during Spanish writing‐to‐dictation. This efficiency allowed the participants to use congruent global information to facilitate performance while avoiding large interference effects from incongruent global information when processing local information, even though suppression of global interference was more evident in monolinguals than in bilinguals.

### Testing the hypothesis of the inhibition of global incongruent information

To test the hypothesis that the reduction of global interference after Spanish writing‐to‐dictation could be interpreted as the suppression or inhibition of global incongruent information during the local task, we performed an additional (a posteriori) analysis on monolinguals and bilinguals. Specifically, we compared the global neutral trials (where the local information does not affect performance) after conflicting local information (where the global incongruent information has been suppressed) with the global neutral trials after neutral local information (in which it is not expected to find suppression of global information).

**FIGURE 6:**
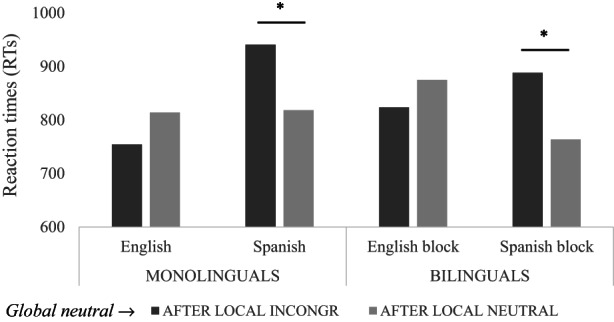
Differences between global neutral trials after local incongruent trials vs. after local neutral trials in monolinguals and bilinguals.

Considering that this additional analysis was planned a posteriori, the number of observations was limited (English monolinguals: after conflicting M = 4.96, after neutral M = 4.17, Spanish monolinguals: after conflicting M = 4.09, after neutral M = 3.77, English block in bilinguals: after conflicting M = 4.56, after neutral M = 3.95, Spanish block in bilinguals: after conflicting M = 4.09, after neutral M = 3.52), and the results should be taken with extreme caution. A repeated measure ANOVA was conducted with the factors of language (Spanish and English), trial (after local incongruent and after local neutral) and their interactions for monolinguals and bilinguals separately (see Figure 6). For monolinguals, the analysis yielded a significant main effect of language, *F* (1, 43) = 4.276, *p* = .045, *η* ^*2*^_*p*_ = .091. In addition, the interaction between language and trial was significant, *F* (1, 43) = 6.112, *p* = .017, *η* ^*2*^_*p*_ = .124, indicating that there were no differences between the two types of trials for English monolinguals (after local incongruent [M = 754.28 ms] vs. local neutral [M = 813.98 ms]; *t* (22) = −1.320, *p* = .200). However, Spanish monolinguals showed slower times for global trials after local incongruent trials (M = 941.04 ms) in comparison with global trials after local neutral trials (M = 818.38 ms); *t* (21) = 2.120, *p* = .046. For bilinguals, the interaction between language and trial was close to significant, *F* (1, 22) = 4.227, *p* = .052, *η* ^*2*^_*p*_ = .168, indicating that there were no differences between the two types of trials after the English block (after local incongruent [M = 823.75 ms] vs. local neutral [M = 874.92 ms]; *t* (22) = −.383, *p* = .705). However, after the Spanish block, the global trials after the local incongruent trials (M = 888.46 ms) were slower in comparison with the global trials after the local neutral trials (M = 763.49 ms); *t* (22) = 2.259, *p* = .039.

Interestingly, the results of Experiments 1 and 2 showed more efficient processing of local information after Spanish writing‐to‐dictation, and we hypothesized that the participants were reducing global interference from the incongruent global information, possibly because they inhibited or suppressed global information. If this hypothesis were true and global information was inhibited, that information would be more difficult to process in a subsequent trial in which it was mandatory (i.e. global neutral). We found that global neutral trials were processed more slowly after local incongruent trials than after local neutral trials and that these differences were restricted to Spanish writing‐to‐dictation in both the monolingual and bilingual groups as evidence of global inhibition during the local task.

## GENERAL DISCUSSION

According to the orthographic depth hypothesis, reading and writing in transparent orthographies are mainly performed through the use of the phonological processing route, in contrast to opaque orthographies, which make extensive use of lexical processing (Frost, 1994; Frost et al., 1987; Katz & Baldasare, 1983; Turvey et al., 1984). In addition, orthographic transparency has been proposed to have an impact on the attentional processing window (grain size of processing; Ziegler & Goswami, 2005) in monolinguals and bilinguals (Adelman et al., 2010; Awadh et al., 2016; Bosse & Valdois, 2009; de León Rodríguez et al., 2015, 2016; Lallier et al., 2013, 2014, 2016, 2018; Lobier et al., 2013; Marzouki & Grainger, 2014; Rau et al., 2014, 2015; Valdois et al., 2014; van den Boer et al., 2013).

However, these theories were proposed for reading acquisition. It is, therefore, mandatory to explore whether the processing dynamics in adults are biased towards lexical or phonological strategies depending on the language transparency, and whether these differences are evident in writing production, where the research remains scarce (Graham et al., 2006). In doing this, we aimed to extend the evidence from reading theories (the orthographic depth hypothesis, the psycholinguistic grain size theory and the grain size accommodation hypothesis) to word writing in monolingual and bilingual adults. In Experiments 1 and 2, we used the writing‐to‐dictation task in Spanish (transparent orthography) or English (opaque orthography), followed by a global–local task to evaluate whether language transparency biased processing towards phonological/lexical processing in writing and to capture the possible influence of language transparency on the visual window. First, we will discuss the results of the writing‐to‐dictation task and the evidence of different strategies of processing (lexical vs. phonological) depending on the degree of language transparency. Second, we will discuss the influence of language on local attention. Finally, we will address the results of the bilingual participants discussing the ability to adapt their processing mode depending on the language transparency.

### Evidence in the writing‐to‐dictation task

According to the assumptions of some reading processing theories (the orthographic depth hypothesis and psychological grain size theory), the writing‐to‐dictation task would be performed differently depending on the transparency of the target language (opaque vs. transparent), which could be evidenced by exploring the effects of certain linguistic variables (Norton et al., 2007). The performance in the rest of the word (the late measure of writing‐to‐dictation) showed a general influence of lexical (frequency and AoA) and phonological (length and neighbours) variables in both languages. Considering that the participants were experienced writers, the lexical and phonological influence on writing was interpreted as evidence of the use of both processing strategies, regardless of language transparency (e.g. Bosse et al., 2003; Folk & Rapp, 2004; Roux & Bonin, 2012; Tainturier et al., 2013).

However, the first access to the word representation during writing‐to‐dictation assessed with performance in the first key performance (Iniesta, Paolieri, et al., 2021; Muscalu & Smiley, 2018) showed a clear dissociation between languages in monolinguals and bilinguals. That is, performance in Spanish was modulated by word length, whereas performance in English was modulated by word frequency and age of acquisition. Importantly, this dissociation was found with classical correlational approaches but also when using Bayesian Pearson correlations (Love et al., 2015). The pattern of effects of these critical variables on the writing‐to‐dictation task is important since they have been previously used to explore lexical and phonological strategies for reading performance in alphabetic scripts (Norton et al., 2007). Indeed, sublexical processing in orthography–phonology conversion systems is modulated by word length (Burani et al., 2007), while lexical processing is associated with the frequency and age of acquisition (Bonin et al., 2004).

Our results provide evidence for the use of two routes or processing strategies in writing, and for the existence of processing biases towards lexical or phonological strategies depending on the transparency of the language in use, extending the evidence from reading theories (the DRC model [Coltheart, 1978; Coltheart et al., 1979, 2001] and the orthographic depth hypothesis [Frost, 1994; Frost et al., 1987; Katz & Baldasare, 1983; Turvey et al., 1984]) to word writing‐to‐dictation (e.g. Bonin & Méot, 2002; Delattre et al., 2006; Ellis & Young, 1988; Rapp et al., 2002).

### Transfer to the global–local letter task

To evaluate the possible attentional modulation depending on the language used in the writing task, we included a global–local letter task (Navon, 1977) that allowed us to explore the facilitating and interfering effects of congruent and incongruent global information on local attention. The results indicated that when the local task was performed after writing‐to‐dictation in Spanish and processing was biased towards the phonological strategy, global information facilitated local attention more than when the task was performed after the English task in both monolinguals and bilinguals. This suggests that, after a Spanish task, participants (Spanish monolinguals and bilinguals in the Spanish block) more efficiently processed local information so that they used the context only if it benefitted performance (the benefit of global congruent information). In addition, after Spanish writing, the participants more efficiently avoided large interference effects from incongruent global information, although suppression of global interference was more evident in monolinguals (Experiment 1) than in bilinguals (Experiment 2), suggesting some language processing differences between the two groups. In contrast, after the English task, participants (English monolinguals and bilinguals in the English block) were less efficient in the local task, as they were not able to benefit from the congruent global context but were hindered by the incongruent global context.

The pattern of transfer effects from the language in use (English or Spanish in the writing‐to‐dictation task) to the attentional task (local or global) can be explained in part by the orthographic depth and grain size hypothesis. The results of the local attention task support the idea that the grain of attentional processing is affected by language transparency. Transparent orthographies are assumed to use smaller processing windows based on fine‐grained phonological features that bias attention towards local features. After the Spanish task, participants seemed to process the local information more efficiently, so they were able to benefit from congruent information and avoid interference from incongruent information. In contrast, when participants performed the local task after English writing, global bias produced less efficient processing in the local task. Opaque orthographies are assumed to use a greater processing grain mainly based on lexical knowledge (e.g. Ziegler & Goswami, 2005) that will bias attention towards global features. This global bias produced by the English writing task would hinder the processing of the information needed to perform the local task, resulting in interference from incongruent information and a lack of facilitation of congruent information. Interestingly, this pattern was evident in monolinguals (Experiment 1) and bilinguals (Experiment 2). Hence, the language differences for the local task can be easily interpreted as suggesting that, after Spanish, local processing is enhanced and proceeds more efficiently.

Despite these nuances, our results show that phonological processing during the Spanish writing‐to‐dictation task (e.g. Coltheart et al., 2001) was transferred to the visual attention task and produced higher local attention efficacy. Thus, the differences in grain size between transparent and opaque orthographies had an impact on attentional windowing (Franceschini et al., 2013; Goswami, 2015; Lallier et al., 2014; Lobier et al., 2013; Onochie‐Quintanilla et al., 2017; Valdois et al., 2014). The differential pattern regarding local attention efficacy in both languages is compatible with the idea of differential processing depending on the language transparency underlying the written production (Brown & Loosemore, 1994; Houghton & Zorzi, 2003; Olson & Caramazza, 1994). This different style of processing affected the windowing or grain sizes (de León Rodríguez et al., 2015; Grainger & Ziegler, 2011), and it was reflected in visual attention patterns (Awadh et al., 2016; Grainger & Ziegler, 2011; Rau et al., 2015) captured with the local task. Interestingly, the differential pattern of use of congruent and incongruent global information during local attention (local efficiency) would seem to reflect the differential influence of (global) lexical information on (local) phonological processing in transparent and opaque orthographies (Lallier & Carreiras, 2018). In transparent orthographies, the result of lexical processing is very similar, and therefore, very congruent to the result of phonological processing, so lexical (global) information would facilitate phonological (local) processing. In contrast, in opaque orthographies, the result of lexical processing is usually incongruent with the results of phonological processing, so lexical (global) information would be hindering phonological (local) processing.

The role of visual attention in reading and writing continues to be the subject of debate (Goswami, 2015), despite the importance of visual attentional skills in literacy acquisition and reading speed. Visual attention has been shown to be a predictor of academic skills (Lobier et al., 2013; Onochie‐Quintanilla et al., 2017; Valdois et al., 2014), and a deficit in visual span has been identified as one of the problems in dyslexia (e.g. Franceschini et al., 2013; Lallier et al., 2014). Evidence for the relationship between global–local attention and phonological and lexical processing has been previously offered by Franceschini et al. (2021), who used the global–local task to bias the grain of attention and explore the influence of lexical and phonological processing within language. Our study is the first extension of the dynamic interaction between language and attention across languages in a writing production task for monolingual and bilingual participants.

### The adaptation of processing strategies to orthographic transparency during bilingual writing

Our results supported the bilingual ability to adapt or modulate processing strategies depending on language opacity (the grain size accommodation hypothesis; Lallier & Carreiras, 2018). That is, participants in the bilingual group replicated the pattern of monolinguals' results. Again, we found evidence of the use of lexical and phonological strategies by experienced writers (e.g. Bosse et al., 2003; Folk & Rapp, 2004; Roux & Bonin, 2012; Tainturier et al., 2013) showing a general influence of lexical (frequency and AoA) but also phonological (length and neighbours) variables in both languages during the later writing stage (rest of the word). However, during the early stage of writing (the first key; Iniesta, Paolieri, et al., 2021; Muscalu & Smiley, 2018), the transparent orthography (i.e. Spanish) biased processing towards phonological processing, whereas the opaque orthography (i.e. English) biased processing towards the lexical strategy (Frost, 1994, 2012; Katz & Frost, 1992; Perfetti & Dunlap, 2008; Ziegler et al., 1997) in the same direction as monolinguals. This pattern provides support for the idea of flexible changes between lexical and sublexical strategies during writing to adapt to the language in use by bilinguals (Sheriston et al., 2016).

In the bilingual group, we replicated the finding that after a Spanish dictation block in which phonological processing was dominant, there was higher local efficacy in the visual attention task. That is, the size of the attentional window seems to vary depending on the grain required to process the language in use (de León Rodríguez et al., 2015, 2016; Iniesta, Paolieri, et al., 2021; Lallier et al., 2013, 2014, 2016; Valdois et al., 2014). Bilinguals showed a similar pattern of facilitation from congruent global information to monolinguals, but they differed in the pattern of interference from incongruent global information. Bilinguals showed similar interference effects from incongruent global information when performing the local task after English or Spanish writing (there was a tendency to reduce interference after Spanish writing). Although the source of this differential effect is not completely clear, it might have to do with cross‐language influences in bilinguals. Numerous studies have provided evidence that linguistic properties of the unintended language affect the production of the intended language at the lexical and phonological levels (Colomé, 2001; Hermans et al., 1998; Macizo & Bajo, 2006; Paolieri et al., 2010). Therefore, co‐activation of the two languages during writing may mitigate the effect of the grain size of the language in use and consequently the strength of the transfer effects from the language to the visual attention task. In addition, as bilinguals use both languages to speak, read, and, in this case, write, it has been suggested that they might have less accumulated literacy practice (see Gollan et al., 2008), resulting in lower local efficiency. Because the pattern of interferences was not as successfully predicted and our explanation is somewhat speculative, further research should be directed towards exploring this hypothesis.

## CONCLUSIONS

The differential effects of the linguistic variables (length, frequency and AoA) in Spanish vs. English writing‐to‐dictation performance support the idea that the degree of transparency was biasing the strategy used during writing (phonological vs. lexical), extending the reading evidence concerning writing‐to‐dictation production to the adult population. In addition, the differential patterns of local efficacy in a subsequent visual attention task led us to conclude that experience with different languages (with different transparencies) modulates the attentional windowing used during processing, supporting the assumptions of reading processing (the DRC model and psychological grain size theory) to writing‐to‐dictation in monolingual and bilingual populations. In addition, the results suggest a bilingual ability to adapt or modulate strategy during processing and to distribute their visual attention according to the transparency of the language in which they are writing.

This research expands the perspective to explore different orthographies following the opacity–transparency continuum (Liu & Cao, 2016; Perfetti & Dunlap, 2008) and different linguistic combinations in bilinguals. One of the limitations of this study is that the bilinguals were unbalanced and Spanish‐dominant. As dominance has been highlighted as an important factor that can affect language processing (e.g. Puig‐Mayenco et al., 2018), it would be especially relevant to explore English‐dominant bilinguals. In addition, it would also be relevant to include bilinguals with different levels of proficiency since this factor might also have an important impact on language processing and language coactivation (e.g. van Hell & Tanner, 2012).

Finally, writing is a complex process involving many sub‐skills, and different paradigms have been proposed to explore word‐writing production. We focused on writing‐to‐dictation, which involved lexical and sublexical processing (Bonin et al., 2015) but with extensive use of transcription skills (see the Simple View of Writing; Berninger et al., 2002). Other forms of writing tasks, such as picture naming or copying (e.g. Afonso et al., 2015; Bonin et al., 2019; Bonin & Fayol, 2000; Damian et al., 2011; Delattre et al., 2006; Quémart & Lambert, 2019; Zhang & Damian, 2010), should be explored to have a more accurate and holistic view of word writing processes, considering that the input characteristics (i.e. visual vs. auditory vs. conceptual) seem to have differential effects on the activation of phonological and orthographic information (see Iniesta, Rossi, et al., 2021). In the same direction, we explored writing‐to‐dictation through a typing paradigm. Although similar processes have been assumed to underlie typing and handwriting (e.g. Pinet et al., 2016; Yamaguchi & Logan, 2014), future research should also directly explore the effect of transparency in handwriting.

## AUTHOR CONTRIBUTIONS

**ANTONIO INIESTA:** Conceptualization; data curation; formal analysis; funding acquisition; investigation; methodology; project administration; resources; software; supervision; validation; visualization; writing – original draft; writing – review and editing. **M.TERESA BAJO:** Conceptualization; data curation; formal analysis; funding acquisition; investigation; methodology; project administration; resources; software; supervision; validation; visualization; writing – original draft; writing – review and editing. **MARTA RIVERA:** Conceptualization; formal analysis; methodology; supervision; visualization. **DANIELA PAOLIERI:** Conceptualization; data curation; formal analysis; funding acquisition; investigation; methodology; project administration; resources; software; supervision; validation; visualization; writing – original draft; writing – review and editing.

## CONFLICT OF INTEREST

All authors declare no conflict of interest.

## Supporting information


Appendix S1

## Data Availability

The data that support the findings of this study are available from the corresponding author upon reasonable request.
