# Supplementary material for: Transfer effects from language processing to visual attention dynamics: The impact of orthographic transparency
Source: Br J Psychol. 2022 Sep 18;114(1):86–111. doi: 10.1111/bjop.12598 (PMC10087185; doi:10.1111/bjop.12598)
Supplement: Supplementary file 1 — Appendix S1 [file BJOP-114-86-s001.docx]

**Supporting Information**

Target words selected in both language blocks (Spanish and English) in the writing to dictation task.

| Spanish block | | | | English block | | | |
| --- | --- | --- | --- | --- | --- | --- | --- |
| octubre | clero | hielo | paz | ability | catalogue | gender | orphanage |
| actriz | conciencia | huérfano | pendiente | absent | champagne | geneva | oyster |
| alcachofa | corona | imperio | prefijo | adjacent | cicada | genius | parsley |
| alucinación | cucaracha | inmigrante | primavera | advantage | circumstance | ginger | penguin |
| árabe | cuerno | jirafa | puente | alive | clover | hawk | price |
| armónica | cuervo | jungla | rango | ambulance | copper | hazelnut | purity |
| aro | diálogo | lasaña | razón | ambush | courgette | headphone | sausage |
| arpa | donativo | lenguaje | reflejo | anchovy | crab | hiccup | seed |
| babero | droga | mano | sabotaje | angle | danger | homage | slavery |
| baile | esponja | margen | salsa | apricot | devil | horizon | sovereign |
| bala | expectativa | martillo | sufijo | avocado | distance | immigration | subjective |
| ballena | fábula | mejillón | tatuaje | barrier | dozen | javelin | sugar |
| banco | flamenco | mensaje | tortuga | beast | drum | jelly | surgeon |
| barbacoa | forraje | monstruo | triángulo | beaver | endive | laxative | tiger |
| barriga | garaje | mostaza | turquesa | bee | evil | legend | tram |
| batalla | gitano | movimiento | vainilla | beret | example | lentil | trumpet |
| berenjena | gobernador | nivel | valiente | bible | excuses | lettuce | varnish |
| bici | gobierno | número | vasco | boat | fever | mobile | vinegar |
| bilingüe | guante | oído | vendaje | bottle | foliage | monk | voice |
| buitre | guía | página | violencia | bright | foreigner | nerve | wolf |
| cacahuete | hambriento | paradoja | yate | bronze | garbage | number | zebra |
| camuflaje | herbívoro | pasajero | zona | bubble | garden | orange | zero |
| celos |  |  |  | caravan |  |  |  |
